# Incidence, predictors, and outcomes of early hospital readmissions after kidney transplantation: Systemic review and meta-analysis

**DOI:** 10.3389/fmed.2022.1038315

**Published:** 2022-11-04

**Authors:** Kinza Iqbal, Muhammad Hasanain, Sawai Singh Rathore, Ayman Iqbal, Syeda Kanza Kazmi, Farah Yasmin, Thoyaja Koritala, Charat Thongprayoon, Salim Surani

**Affiliations:** ^1^Department of Internal Medicine, Dow University of Health Sciences, Karachi, Pakistan; ^2^Department of Internal Medicine, Dr. Sampurnanand Medical College, Jodhpur, Rajasthan, India; ^3^Department of Internal Medicine, Mayo Clinic Health System, Mankato, MN, United States; ^4^Division of Nephrology and Hypertension, Mayo Clinic, Rochester, MN, United States; ^5^Department of Pulmonology, Texas A&M University College of Medicine, Bryan, TX, United States; ^6^Department of Anesthesiology, Mayo Clinic, Rochester, MN, United States

**Keywords:** readmission, early hospital readmission, kidney transplant, incidence, predictors

## Abstract

**Background:**

Early hospital readmission (EHR) within 30 days after kidney transplantation is a significant quality indicator of transplant centers and patient care. This meta-analysis aims to evaluate the incidence, predictors, and outcomes of EHR after kidney transplantation.

**Methods:**

We comprehensively searched the databases, including PubMed, Cochrane CENTRAL, and Embase, from inception until December 2021 to identify studies that assessed incidence, risk factors, and outcome of EHR. The outcomes included death-censored graft failure and mortality. Data from each study were combined using the random effect to calculate the pooled incidence, mean difference (MD), odds ratio (OR), and hazard ratio (HR) with 95% confidence interval (CI).

**Results:**

A total of 17 studies were included. The pooled EHR incidence after kidney transplant was 24.4% (95% CI 21.7–27.3). Meta-analysis showed that recipient characteristics, including older recipient age (MD 2.05; 95% CI 0.90–3.20), Black race (OR 1.31; 95% CI 1.11, 1.55), diabetes (OR 1.32; 95% CI 1.22–1.43), and longer dialysis duration (MD 0.85; 95% CI 0.41, 1.29), donor characteristics, including older donor age (MD 2.02; 95% CI 0.93–3.11), and transplant characteristics, including delayed graft function (OR 1.75; 95% CI 1.42–2.16) and longer length of hospital stay during transplantation (MD 1.93; 95% CI 0.59–3.27), were significantly associated with the increased risk of EHR. EHR was significantly associated with the increased risk of death-censored graft failure (HR 1.70; 95% CI 1.43–2.02) and mortality (HR 1.46; 95% CI 1.27–1.67) within the first year after transplantation.

**Conclusion:**

Almost one-fourth of kidney transplant recipients had EHR within 30 days after transplant, and they had worse post-transplant outcomes. Several risk factors for EHR were identified. This calls for future research to develop and implement for management strategies to reduce EHR in high-risk patients.

## Introduction

Kidney transplantation is the best renal replacement therapy option for end-stage kidney disease patients. Kidney transplant recipients have a higher long-term survival and quality of life than those who remains on dialysis (1, 2). Despite the advances in kidney transplantation and post-transplant care, hospital readmission is still frequent. Kidney transplant recipients are at higher risk of readmission given more comorbidity burden and vulnerability to complications (3, 4).

Early hospital readmission (EHR), defined as any hospitalization within 30 days of discharge following kidney transplantation, is a significant quality indicator of transplant centers and patient care (5). EHR is related to an increased morbidity, decreased quality of life, and higher medical expenditure and resource utilization (6). Reduced reimbursements from Medicare for hospitals with higher-than-expected readmission rates have been implemented due to recent policy changes aimed to reduce avoidable hospital readmissions and to improve health outcomes while reducing medical expenditure (6, 7). The incidence of EHR after kidney transplantation reported in the literature is variable. Different risk factors for EHR after kidney transplantation have been described (5, 8–10). Recognizing the risk factors for EHR is critical for identifying kidney transplant recipients who may benefit from additional post-transplant surveillance and the development of new strategies to reduce EHR.

The objective of this meta-analysis was to determine the incidence of EHR, identify the risks factors for EHR, and assess the impact of EHR on post-transplant outcomes in kidney transplant recipients.

## Materials and methods

This article has been reported in accordance with the Preferred Reporting Items for Systematic Reviews and Meta-Analysis (PRISMA) guidelines (11).

### Data sources and search strategy

We conducted a systematic literature search for relevant articles in the databases, including Pubmed, Embase, and Cochrane CENTRAL, using a comprehensive search strategy from inception until December 20th, 2021. The combination of the following MeSH keywords was used: “renal transplant,” “kidney transplant,” “readmission,” “early hospital readmission,” “30-day readmission,” “incidence,” “rate,” “predictor,” “risk factors,” and “association.” The detailed search strategy is presented in Supplementary Table 1.

### Study selection and inclusion criteria

We included studies that reported the incidence, predictors, or outcomes of EHR after kidney transplantation. EHR was defined as 30-day readmission to any institution, due to any cause, after kidney transplantation. We excluded studies with (1) readmission >30 days after kidney transplantation, (2) combined kidney transplantations with other organs, (3) no outcomes of interest, and (4) reviews and letters. Duplicated studies retrieved from the systemic search were identified and eliminated using Endnote (Clarivate Analytics, Thomson Reuters Corporation, Philadelphia, Pennsylvania). The articles were screened based on titles and abstracts by two independent researchers (KI, AI) and subsequently assessed for relevance by reviewing full-text articles. References of the articles were also screened to identify additional studies.

The potential of sample dependence arises when multiple papers included in the review report findings from analyses on the same cohort of patients. When the studies had overlapping periods, the potential for sample dependence was minimized by the selection of studies with the longest period of data collection as the representative study for that cohort, for each variable. However, when two or more studies had the same period of data collection, the study with higher methodological quality was selected as the representative study.

### Data extraction and outcomes

Two independent researchers (KI and AI) extracted data from the eligible articles. The following information was extracted: name of the first author, year of publication, study design, country of origin, sample size, subject demographics, comorbidities, and incidence of EHR. Data regarding the risk factors for EHR included the following recipient characteristics: age, gender, black race, body mass index (BMI), diabetes, prior dialysis, and dialysis duration; donor characteristics: age, donor type, and expanded donor criteria; and transplant characteristics: delayed graft function (DGF) and length of hospital stay during transplantation. The outcomes of EHR included death-censored graft failure and mortality within 1 year after kidney transplant. Raw data and adjusted estimates were extracted.

For studies that provided medians and ranges instead of means and standard deviations or provided only means in the absence of standard deviations, the means and/or standard deviations were calculated using the formula described by Hozo et al. (12). Some studies reported readmissions within 30 days of the procedure and others reported those within 30 days of discharge, readmissions data were extracted according to either definition, and the definition used by each study was recorded.

### Statistical analysis and quality assessment

We utilized Review Manager v.5.3 (The Nordic Cochrane Center, The Cochrane Collaboration, 2014) and MedCalc v 20.027 to perform all the analyses. A random-effects model was used to calculate the Mantel Haenszel odds ratios (OR) for dichotomous variables and mean difference (MD) for continuous variables. Adjusted estimates were reported using inverse variance adjusted Hazard ratios (aHR). All estimates were reported with a confidence interval (CI) of 95% and a *p*-value < 0.05 was considered significant in all cases. We examined the correlation between the risk factors and EHR; risk factors reported in 3 or more studies were statistically analyzed. When available, risk factors based on multivariate analysis were also collected. To rule out the possibility of any single study disproportionately affecting the results, a leave-one-out sensitivity analysis was carried out by removing one study at a time. The quality appraisal of the included studies was performed by using the Newcastle–Ottawa Quality Assessment Scale (13). Each study was graded as: low bias risk (8–9 points), moderate bias risk (5–7 points), or significant bias risk (0–4 points).

## Results

### Literature search and baseline characteristics

**Figure 1:**
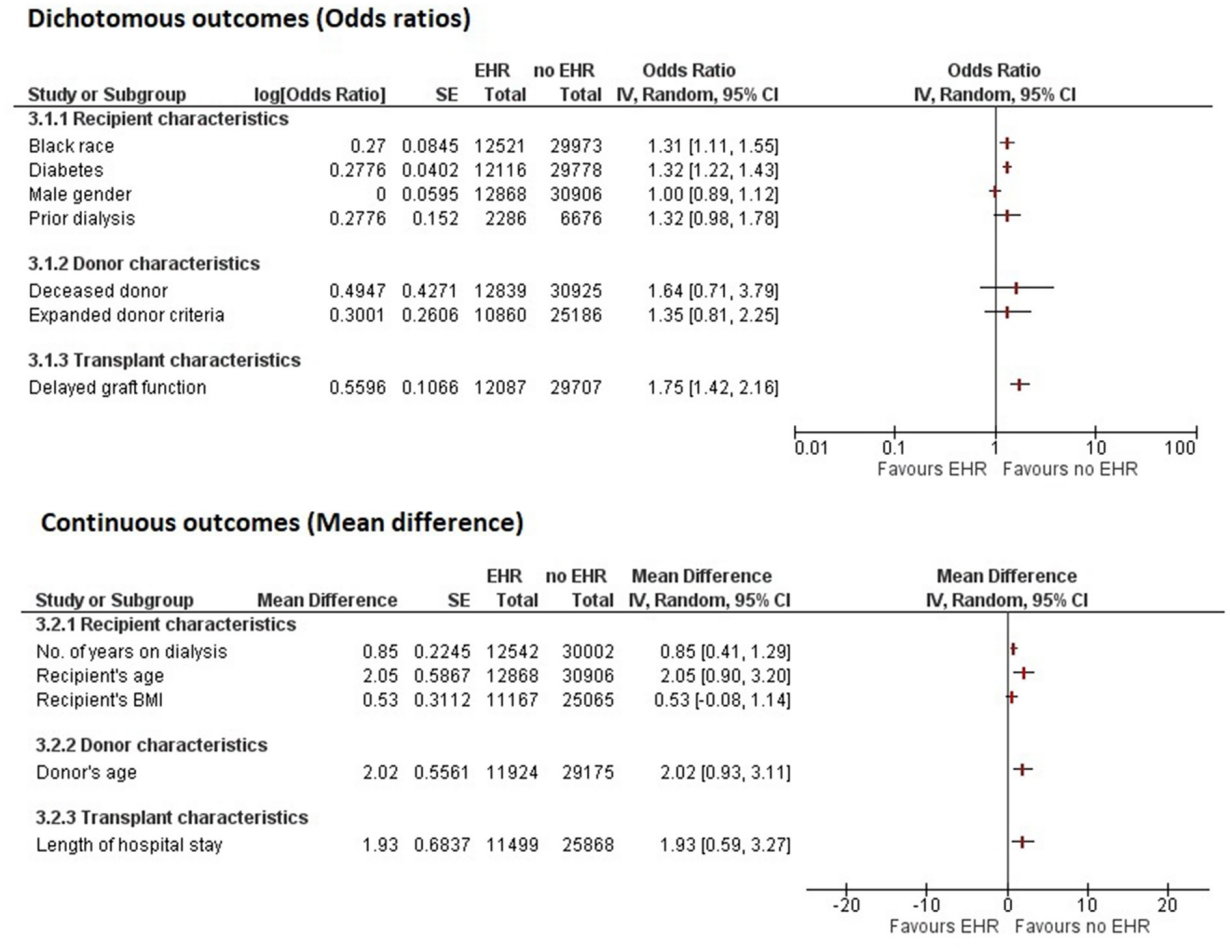
Forest plot summarizing the pooled analyses of all potential factors associated with early hospital readmission (30-day) after kidney transplantation. IV, inverse variance; SE, standard error; CI, confidence interval; HER, early hospital readmission.

The initial search strategy identified a total of 700 potentially relevant articles. After excluding the duplicates, 408 articles were screened for relevance based on their titles and abstracts. Out of these, 52 full-text articles that aligned with the objective of the manuscript were reviewed. Ultimately, 17 studies were included in the final analysis, out of which twelve were cohort studies (10 retrospective, 1 prospective, and 1 ambispective) and one was a case-control study (5, 8–10, 14–25). Supplementary Figure 1 presents the PRISMA flowchart outlining the search process. Table 1 summarizes the study characteristics of the included articles and the causes of EHR. The results of the meta-analysis of potential risk factors for EHR are presented in Table 2. Figure 1 illustrates the results of all pooled analyses, while Supplementary Figures 2–19 present the individual plots of each potential risk factor of EHR.

**Table 1:** Baseline characteristics of included studies.

**Study**	**Country**	**Study design**	**Total participants**	**No. of readmitted patients**	**No. of non-readmitted patients within 30 days**	**Readmission rate**	**Definition of readmission**	**Age [mean (SD)]**	**Male gender (%)**	**Causes of 30-day readmission**
Bergman et al. (8)	Canada	Retrospective cohort; single-center	213	41	172	19.20%	30-days readmission rate	–	67.6	Renal (36.6%), infectious (29.3%), and gastrointestinal issues (21.9%)
Chu et al. (9)	China	Retrospective chart review; single-center	518	9	509	1.74%	30-days readmission rate	33.75	71	–
Covert et al. (14)	US	Retrospective case-control; single-center	384	64	320	16.70%	30-days readmission rates in kidney transplant recipients.		54.4	Infection (19%), surgical (18%), surgical complications (18%), Others (15%)
Dols et al. (15)	US	Retrospective, observational study	315	70	245	22.20%	Hospital readmissions within 30 days following kidney transplantation	–	–	Graft dysfunction (46%), nausea/vomiting (18%), infection (18%), volume overload or depletion (15%), and surgical complications (13%).
Famure et al. (16)	US	Ambispective observational cohort; single-center	1,093	212	881	19.40%	First re-admission occurring within 30 days after discharge from the transplant hospitalization.	49.9 (13.2)	61.1	Infection (21), renal and genitourinary (20.5), rejection (14.9), drug toxicity (8.3), surgical complication (7.4), cardiovascular (5.2), gastrointestinal (1.8), endocrine (0.9), other (18.3)
Hogan et al. (17)	US	Retrospective cohort; multi-center	40,461	12,985	27,476	31.80%	Hospital readmission within 30 days of discharge from transplant hospitalization	53.12 (13.68)	61.99	–
Kang et al. (18)	Korea	Retrospective, observational study; single-center	103	32	71	31.10%	1 or more readmissions within 30 days	–	62.1	Electrolyte imbalance (46.9%), acute rejection (18.6%), surgical complications (9.4%), infection
Kim et al. (5)	UK	Prospective cohort; single-center	269	56	213	20.82%	≥1 hospital readmission within 30 days of discharge from transplant hospitalization	Median 55 (41–64)	59.11	Surgical reasons (25%): lymphocele, urinoma, hematoma, hernia, and infected incision site; infectious (18%): transplant pyelonephritis, neutropenic fever, pneumonia, cellulitis, gastroenteritis; metabolic (18%): electrolyte abnormalities, altered mental status; renal (14%): acute kidney injury (AKI) due to acute tubular necrosis (ATN) or acute rejection; gastrointestinal (12.5%); cardiovascular (9%); and miscellaneous (3.5%): anxiety, autonomic dysfunction.
Lichvar et al. (19)	US	Retrospective cohort study; single-center	216	71	145	32.80%	30-day readmission rate	50.5 (SD 13.9)	60.7	Electrolyte abnormalities (18.3%), allograft dysfunction (12.0.7)
Luan et al. (20)	US	Retrospective cohort study; single-center	1,064	286	778	26.90%	Hospital readmissions within 30 days following kidney transplantation	49.3 (13.2)	62.8	Surgical complications (32.4%), infection (20.1%), acute kidney injuries/acute rejection (13.0%), Cardiovascular (11.0%), fluid and electrolyte issues (11.5%), gastrointestinal complaints (5.7%), deep vein thrombosis (1.3%), and others (5.0%).
Lubetzky et al. (21)	US	Retrospective cohort study; single-center	462	145	317	31.40%	≥1 hospital readmission within 30 days of discharge from transplant hospitalization		60.2	Surgical (20.7%), infection (21.7%), graft dysfunction (20.9%), gastrointestinal (21.7%), metabolic (21.7%), and others (13.9%)
McAdams-Demarco et al. (10)	US	National study of longitudinal Medicare claims data; multicenter	32,961	10,052	22,909	31%	≥1 hospital readmission within 30 days of discharge from transplant hospitalization	47.5	41	Renal (36), infection (12), endocrine (11), gastrointestinal (7), circulatory (6), allergy or drug effects (3), trauma (3), rehabilitation (3), renal failure (2), and others (17)
Naylor et al. (22)	Canada	Population-based cohort; multi-center	5,437	1,128	4,309	20.70%	Hospital readmission within 30 days of discharge from transplant hospitalization		36.6	Rejection (18.7%); complications of procedures, not elsewhere classified (13.6%); acute renal failure (5.7%); other disorders of urinary system (4.3%); and post-procedural disorders of genitourinary system, not elsewhere classified (2.6%)
Nguyen et al. (23)	US	Retrospective cohort study; single-center	2,371	749	1,622	32%	≥1 hospital readmission within 30 days of discharge from transplant hospitalization	median 50	60	Graft dysfunction (26.9%), gastrointestinal (16.3%), infection (11.2%), fluid and electrolyte abnormalities (9.3%), fever evaluation (8.7%), and hematologic (4.8%), pulmonary (4.1%), cardiovascular (4.6%), urologic (3.3%), surgical (3%)
Schucht et al. (24)	US	Retrospective chart review; single-center	141	37	104	26.20%	30-day readmission rate	54.8 (13.7)	55	–
Tavares et al. (25)	Brazil	Retrospective cohort study; single-center	1,175	313	862	26.60%	Hospital readmission within 30 d following kidney transplantation	45.9 (35.2–54.5)	62.6	Infection (67%), surgical complications (14%), metabolic disturbances (11%), acute rejection (4.8%), cardiovascular events (2.2%), and renal artery stenosis (1%)
Whitlock et al., (26)	US	Retrospective chart review; single-center	325	99	226	30.46%	Hospital readmission within 30 days of discharge from transplant hospitalization	52.3 (42.8, 61.1)	60.9	–

SD: standard deviation.

**Table 2:** Meta-analysis of the risk factors and outcomes associated with early hospital readmission (30-day) after kidney transplantation.

**Potential associations**	**No. of studies**	**No. of participants**	**Pooled estimates**	**Lower limit 95% CI**	**Upper limit 95% CI**	***p*-value**	**Heterogeneity *I*^2^ (%)**
**Recipient characteristics**							
Age	9	43,774	MD: 2.05	0.90	3.20	0.0005^*^	97
Gender	9	43,774	OR: 1.00	0.89	1.12	0.98	56
Black race	7	42,494	OR: 1.31	1.11	1.55	0.001^*^	64
Body mass index	4	36,232	MD: 0.53	−0.08	1.14	0.09	77
Diabetes	8	41,894	OR: 1.32	1.22	1.43	< 0.00001^*^	14
Prior dialysis	5	8,962	OR: 1.32	0.98	1.78	0.07	57
Number of years on dialysis	7	42,544	MD: 0.85	0.41	1.29	0.0001^*^	99
**Donor characteristics**							
Age	5	41,099	MD: 2.02	0.93	3.11	0.0003^*^	96
Status of the donor (alive/dead)	9	43,764	OR: 1.64	0.71	3.79	0.24	99
Expanded donor criteria	5	36,046	OR: 1.35	0.81	2.25	0.25	93
**Transplant characteristics**							
Delayed graft function	7	41,794	OR: 1.75	1.42	2.16	< 0.00001^*^	82
Length of hospital stay during transplantation	5	37,367	MD: 1.93	0.59	3.27	0.005^*^	99
**Outcomes associated with EHR**							
Death-censored graft failure within the first year after transplantation.	3	6,754	HR: 1.70	1.43	2.02	< 0.00001^*^	2
Mortality within the first year of renal transplant	3	6,754	HR: 1.46	1.27	1.67	< 0.00001^*^	0

OR, odds ratio; MD, mean difference; HR, hazard ratio; CI, confidence interval; p-value: probability value,

* significant.

### Quality assessment and publication bias

The methodological quality assessment of included studies (Supplementary Table 2) showed that seven studies had low risk of bias, while 10 had moderate risk of bias. Therefore, all the studies were eligible for quantitative analysis. The funnel plots of publication bias are illustrated in Supplementary Figures 20, 21. There was no significant publication bias among all the outcomes, and the individual *p*-values of Begg-Mazumdar's rank correlation test and Egger's regression test are presented in Supplementary Table 3.

### Results of meta-analysis

#### Incidence of early hospital readmission

**Figure 2:**
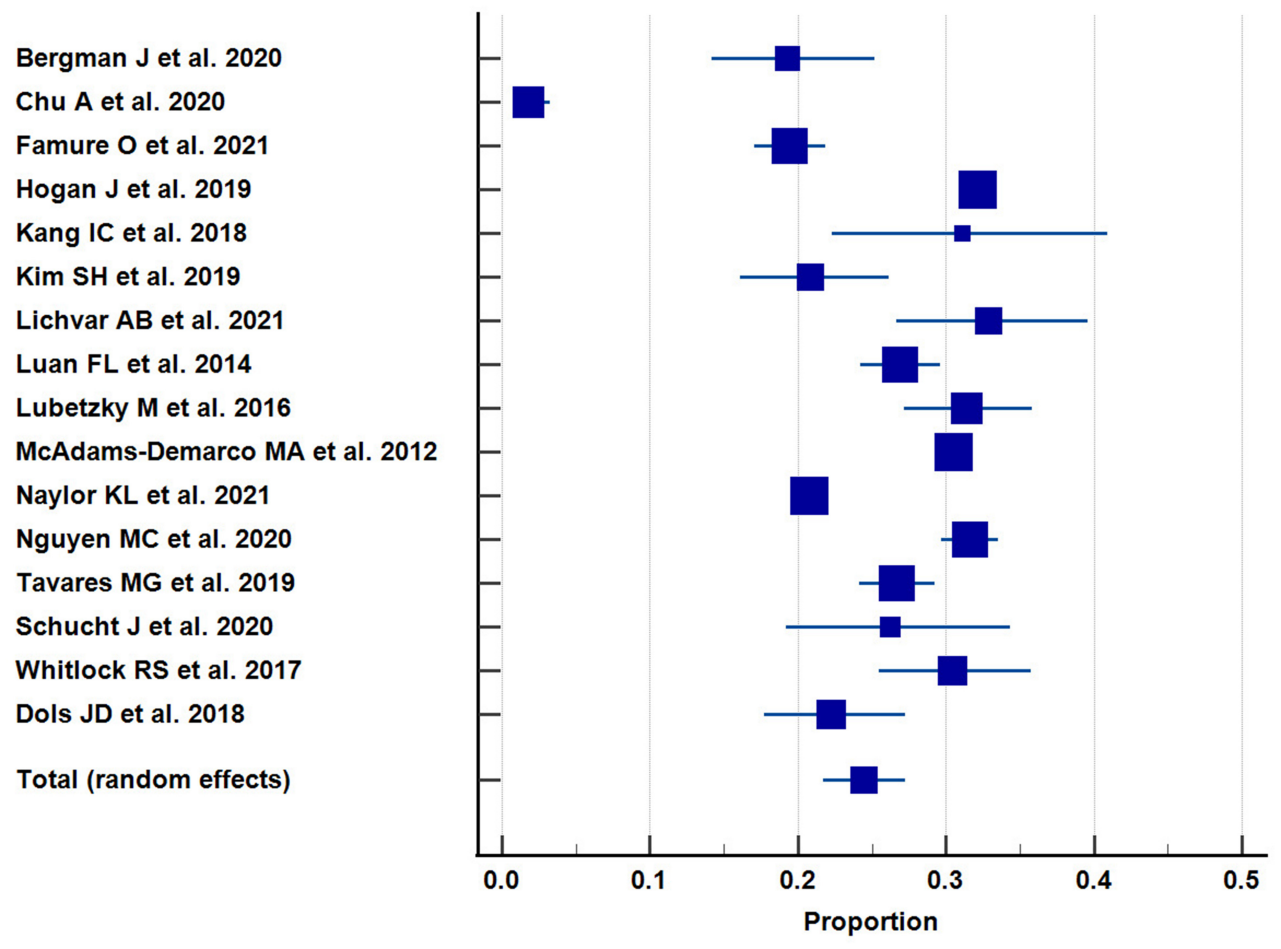
The pooled incidence of 30-day readmission after kidney transplantation.

A total of 16 studies reported the incidence of EHR after kidney transplantation in 26,285 out of total 87,124 transplant recipients. The pooled incidence of EHR in kidney transplant recipients was 24.4% [95% CI = 21.7–27.3 %; *I*^2^ = 98.26%; Figure 2).

#### Predictors

##### Recipient characteristics

Recipient characteristics assessed across included studies were age, gender, black race, body mass index, diabetes, prior dialysis, and number of years on dialysis. Meta-analysis revealed that recipient's older age (MD = 2.05 [95% CI 0.90, 3.20]; *p* = 0.0005; *I*^2^ = 97%), black race (OR = 1.31 [95% CI 1.11, 1.55]; *p* = 0.001; *I*^2^ = 64%), diabetes (OR = 1.32 [95% CI 1.22, 1.43]; *p* < 0.00001; *I*^2^ = 14%), and longer dialysis duration (MD = 0.85 [95% CI 0.41, 1.29]; *p* = 0.0001; *I*^2^ = 99%) were significantly associated with increased EHR (Figure 1, Supplementary Figures 2–5). However, no significant association of EHR was found with recipient's gender (OR = 1.00 [95% CI 0.89, 1.12]; *p* = 0.98; *I*^2^ = 56%), body mass index (MD = 0.53 [95% CI −0.08, 1.14]; *p* = 0.09; *I*^2^ = 77%), and prior dialysis (OR = 1.32 [95% CI 0.98, 1.78]; *p* = 0.07; *I*^2^ = 57%) (Figure 1, Supplementary Figures 6–8).

On pooling studies that reported adjusted data, we observed that recipient's older age (MD = 1.16 [95% CI 1.00, 1.35]; *p* = 0.05; *I*^2^ = 89%) and longer dialysis duration (MD = 1.01 [95% CI 1.00, 1.02]; *p* = 0.04; *I*^2^ = 76%) remained significantly associated with EHR (Supplementary Figures 9, 10).

##### Donor characteristics and transplant characteristics

The meta-analyzed donor characteristics included older age, donor type, and expanded donor criteria. Older donor age was significantly associated with increased EHR (MD = 2.02 [95% CI 0.93, 3.11]; *p* = 0.0003; *I*^2^ = 96%) (Figure 1, Supplementary Figure 11). However, deceased donor (OR = 1.64 [95% CI 0.71, 3.79]; *p* = 0.24; *I*^2^ = 99%) and expanded donor criteria (OR = 1.35 [95% CI 0.81, 2.25]; *p* = 0.25; *I*^2^ = 93%) were not significantly associated with EHR (Figure 1, Supplementary Figures 12, 13).

Transplant characteristics, including delayed graft function (OR = 1.75 [95% CI 1.42, 2.16]; *p* < 0.00001; *I*^2^ = 82%) and longer length of hospital stay during transplantation (MD = 1.93 [95% CI 0.59, 3.27]; *p* = 0.0003; *I*^2^ = 99%), were significantly associated with increased EHR (Figure 1, Supplementary Figures 14, 15). On adjusted analysis, delayed graft function (aOR = 1.43 [95% CI 1.11, 1.85]; *p* = 0.006; *I*^2^ = 74%) and longer length of hospital stay (aOR = 1.20 [95% CI 1.07, 1.36]; *p* = 0.002; *I*^2^ = 91%) and remained significantly associated with increased EHR (Supplementary Figures 16, 17).

The results of leave-one-out sensitivity analysis and studies that caused a significant drop in heterogeneity are shown in Supplementary Table 4. Deceased donor (OR = 1.35 [0.81, 2.25]; *p* = 0.0010; *I*^2^ = 68%) and expanded donor criteria (OR = 1.60 [1.11, 2.32]; *p* = 0.01; *I*^2^ = 66%) became significant predictors of EHR on performing leave-one-out sensitivity analysis.

#### Outcomes

Overall, three studies with a total of 1,460 kidney transplant recipients with EHR and 5,294 recipients without EHR documented the association of EHR with death-censored graft failure and mortality within 1 year after kidney transplant. EHR was significantly associated with increased risk of death-censored graft failure within the first year after transplantation (aHR = 1.70 [95% CI 1.43, 2.02]; *p* < 0.00001; *I*^2^ = 2%) (Supplementary Figure 18). EHR was significantly associated with increased mortality (aHR = 1.46 [95% CI 1.27, 1.67]; *p* < 0.00001; *I*^2^ = 0%) (Supplementary Figure 19).

## Discussion

In the current meta-analysis, we have summarized pertinent evidence on the incidence, risk factors, and outcomes of EHR in kidney transplantation. Significant recipient-related risk factors of EHR after kidney transplantation included age, gender, black race, BMI, diabetes, and a higher number of years on dialysis. Similarly, older donor age and deceased donor were significant donor-related predictors of EHR. Delayed graft function (DGF) and a longer length of hospital stay during transplantation were significant transplant characteristics that increased the odds of EHR. Moreover, EHR was significantly associated with incident death-censored graft failure and mortality within the first year of transplantation.

Our study showed a pooled incidence of 30-day readmission of 24.4% [95% CI = 21.7–27.3 %). This is higher than the incidence of readmission previously reported in patients undergoing orthopedic procedures (5.4%), colectomy (14.7%), and pancreatic resection (19.1%) (27–29). However, other organ transplantation studies on liver (30.6%) and lung transplantation (45.4%) have reported higher incidence of EHR (30, 31).

We found an increased risk of EHR in black recipients. This finding is consistent with a greater risk of readmission in black recipients in conditions such as congestive heart failure, myocardial infarction, and pneumonia (32). Moreover, a longer time on dialysis was observed to be a significant risk factor for EHR in kidney transplantation. This could be explained by the immunological modifications, associated comorbidity burden, and physiological reserve decline (17, 33). An increase in risk of EHR and mortality due to infections have been shown in both hemodilaysis and peritoneal dialysis patients (34). Meier-Kriesche et al. reported that a longer duration of dialysis pre-transplant was associated with an increased risk of death censored graft loss (*p* < 0.001). Dialysis treatment of 6–12, 12, and 12–24 months was associated with a 37, 55, and 68% greater risk for death-censored graft loss, respectively (35).

In our analysis diabetes increased the risk of EHR. A previous retrospective study of 366 kidney transplant, transplant due to diabetic nephropathy was significantly associated with more and earlier post-transplant readmissions compared with patients who underwent transplants due to non-diabetic end-stage kidney disease (36). Diabetes is one of the most important factors for recurrent urinary tract infection after the transplant, and these types of infections are the most frequent in renal transplant patients (37). Moreover, Enomoto et al. (38) reported that diabetic patients were more likely to be readmitted (adjusted OR = 1.17, 95% CI 1.15–1.19; *p* < 0.001) compared to non-diabetics. Factors associated with readmissions included infections (9.4 vs. 7.7%), heart failure (6.0 vs. 3.1%), and chest pain/myocardial infarction (5.5 vs. 3.3%) (39).

DGF leads to an increased risk of EHR and short-term as well as long-term graft loss (39). Dialysis-dependent states and various other comorbidities are also linked with a longer length of hospital stay. Prolonged hospital stay could lead to higher chances of contracting infections. A shorter length of stay may indicate a low-risk recipient receiving a kidney from a low-risk donor (19). EHR was also significantly associated with death-censored graft failure and mortality within the first year of kidney transplant. Heldal et al. showed that DGF was an independent risk factor for death-censored graft loss in patients aged 60 years or more, while Faravardeh et al. reported that DGF and acute rejection were predictors for graft failure in younger recipients as well (40, 41). Mortality is associated with infectious, cardiovascular, and cerebrovascular complications but also depends on transplant center practices and the quality of post-transplant follow-up (5).

Our systematic review suggests patients at high risk of EHR can be identified through relevant risk factors. Patients are likely to have more than one of the above-mentioned risk factors and, therefore, predictive models should be developed to identify patients at high risk of EHR at the time of discharge. It may provide the basis of a robust risk predictive model given the number of studies included. Such patients could be selected in clinical trials to experiment with interventions to prevent early readmissions. A systematic review by Leppin et al. observed that tested interventions prevented 30-day readmissions in patients admitted to an inpatient ward for a minimum of 24 h for any medical or surgical reason. They reported that multidisciplinary strategies which increase patients' easy access to post-discharge care were the most successful (42). Quality improvement initiatives decreased the risk of readmissions by 23.7, 12.1, and 6.3% in chronic obstructive pulmonary disease, congestive heart failure patients and the general population, respectively (43, 44). Taber et al. implemented a multi-faceted strategy to improve health care value for kidney transplant patients, particularly those who developed DGF. They reported that the length of hospital stay during transplantation in DGF patients decreased from 8 to 4 days at the start of the intervention, while the national length of hospital stay during this time was 10 days (45). Moreover, focus on patient education, improved discharge planning, post-discharge phone calls, patient hotlines, and follow-up home visits also yielded positive results (46).

There are several limitations in our meta-analysis. First, most of the included articles were single-center observational studies, which limits cohort size and generalization of data. Most studies were retrospective in nature; therefore, the inherent confounding of the study type was inevitable. Second, some included articles defined EHR as readmission within 30 days from the date of the transplant procedure, while others measured EHR from the date of discharge after transplantation. Third, our study reported significant heterogeneity across various risk factors. Heterogeneity in our meta-analysis could be due to various factors that lead to effect size variability. The high I-squared values in the incidence of EHR and other risk factors in our study could be attributed to differences in sex, age, surgeon training, and time from enrollment in the included studies. Last, there was insufficient data to assess the role of type of immunosuppression on EHR after kidney transplantation.

## Conclusion

This meta-analysis reported a high incidence of EHR in kidney transplant patients and summarized the evidence available on the risk factors associated with it. The most prominent risk factors include recipient's black race, diabetes, a higher number of years on dialysis, delayed graft function (DGF), and a longer length of hospital stay during transplantation. EHR is associated with death censored graft failure and mortality within the first year of transplantation. Hence, future research should aim to develop and implement predictive models for patient identification and novel management strategies to reduce EHR in patients at risk.

## Data availability statement

Publicly available datasets were analyzed in this study. This data can be found here: Data is available upon reasonable request from the authors.

## Author contributions

KI and SSR: concept/design. AI and SKK: data analysis/interpretation. MH, KI, AI, and SKK: drafting article. FY, TK, CT, and SS: critical revision of the article. TK, CT, and SS: approval of the article. FY: statistics. AI and SSR: data collection. All authors contributed to the article and approved the submitted version.

## Conflict of interest

The authors declare that the research was conducted in the absence of any commercial or financial relationships that could be construed as a potential conflict of interest.

## Publisher's note

All claims expressed in this article are solely those of the authors and do not necessarily represent those of their affiliated organizations, or those of the publisher, the editors and the reviewers. Any product that may be evaluated in this article, or claim that may be made by its manufacturer, is not guaranteed or endorsed by the publisher.

## Abbreviations

EHR: early hospital readmission
DGF: delayed graft function.
