# Supplementary material for: Incidence, predictors, and outcomes of early hospital readmissions after kidney transplantation: Systemic review and meta-analysis
Source: Front Med (Lausanne). 2022 Nov 4;9:1038315. doi: 10.3389/fmed.2022.1038315 (PMC9672339; doi:10.3389/fmed.2022.1038315)
Supplement: Supplementary file 1 [file Data_Sheet_1.docx]

**Supplementary Table 1: Detailed search strategy.**

| **Electronic database** | **Detailed search strategy** |
| --- | --- |
| Pubmed | (("kidney transplantation"[MeSH Terms] OR ("kidney"[All Fields] AND "transplantation"[All Fields]) OR "kidney transplantation"[All Fields] OR ("renal"[All Fields] AND "transplant"[All Fields]) OR "renal transplant"[All Fields] OR ("kidney transplantation"[MeSH Terms] OR ("kidney"[All Fields] AND "transplantation"[All Fields]) OR "kidney transplantation"[All Fields] OR ("kidney"[All Fields] AND "transplant"[All Fields]) OR "kidney transplant"[All Fields])) AND ("readmission"[All Fields] OR "readmissions"[All Fields] OR ("early"[All Fields] AND ("patient readmission"[MeSH Terms] OR ("patient"[All Fields] AND "readmission"[All Fields]) OR "patient readmission"[All Fields] OR ("hospital"[All Fields] AND "readmission"[All Fields]) OR "hospital readmission"[All Fields])) OR ("patient readmission"[MeSH Terms] OR ("patient"[All Fields] AND "readmission"[All Fields]) OR "patient readmission"[All Fields] OR "30 day readmission"[All Fields])) AND ("epidemiology"[MeSH Subheading] OR "epidemiology"[All Fields] OR "incidence"[All Fields] OR "incidence"[MeSH Terms] OR "incidences"[All Fields] OR "incident"[All Fields] OR "incidents"[All Fields] OR ("j rehabil assist technol eng"[Journal] OR "rate"[All Fields]) OR ("predictor"[All Fields] OR "predictors"[All Fields]) OR ("risk factors"[MeSH Terms] OR ("risk"[All Fields] AND "factors"[All Fields]) OR "risk factors"[All Fields]) OR ("associate"[All Fields] OR "associated"[All Fields] OR "associates"[All Fields] OR "associating"[All Fields] OR "association"[MeSH Terms] OR "association"[All Fields] OR "associations"[All Fields]))) |
| Embase | (renal transplant OR kidney transplant) AND (readmission OR early hospital readmission OR 30-day readmission) AND (incidence OR rate OR predictor OR risk factors OR association) |
| Cochrane | (renal transplant OR kidney transplant) AND (readmission OR early hospital readmission OR 30-day readmission) AND (incidence OR rate OR predictor OR risk factors OR association) |

**Supplementary Table 2: Assessment of methodological quality of the included studies using Newcastle Ottawa scale**

| **Study** | **SELECTION** | | | | COMPARABILITY | **OUTCOME** | | | Total (maximum = 9) |
| --- | --- | --- | --- | --- | --- | --- | --- | --- | --- |
|  | Representativeness of the exposed cohort | Selection of the non- exposed cohort | Ascertainment of the exposure | Outcome status at start of study |  | Assessment of the outcome | Length of follow-up | Adequacy of follow-up |  |
| Bergman J et al. 2020 | * | * | * | * | * | * | * | * | 8 |
| Chu A et al. 2020 | * | * | * | - | - | * | * | * | 6 |
| Covert KL et al. 2016 | * | * | * | * | * | * | * | * | 8 |
| Dols JD et al. 2018 | * | * | * | - | * | * | * | * | 7 |
| Famure O et al. 2021 | * | * | * | * | * | * | * | * | 8 |
| Hogan J et al. 2019 | * | * | * | * | * | * | * | * | 8 |
| Kang IC et al. 2018 | * | * | * | - | * | * | * | * | 7 |
| Kim SH et al. 2019 | * | * | * | * | * | * | * | * | 8 |
| Lichvar AB et al. 2021 | * | * | * | * | * | * | * | * | 8 |
| Luan FL et al. 2014 | * | * | * | - | * | * | * | * | 7 |
| Lubetzky M et al. 2016 | * | * | * | - | - | * | * | * | 6 |
| McAdams-Demarco MA et al. 2012 | * | * | * | - | * | * | * | * | 7 |
| Naylor KL et al. 2021 | * | * | * | - | * | * | * | * | 7 |
| Nguyen MC et al. 2020 | * | * | * | - | - | * | * | * | 6 |
| Schucht J et al. 2020 | * | * | * | * | * | * | * | * | 8 |
| Tavares MG et al. 2019 | * | * | * | - | * | * | * | * | 7 |
| Whitlock RS et al. 2017 | * | * | * | - | * | * | * | * | 7 |

**Supplementary Table 3: Publication bias for potential risk factors.**

| **Risk factor** | **Bias Egger** | **Bias Begg** |
| --- | --- | --- |
| **Recipient characteristics** | | |
| Age | 0.2497 | 0.8348 |
| Gender | 0.4498 | 0.0606 |
| Black race | 0.5947 | 0.8806 |
| Body mass index | 0.7535 | 0.1742 |
| Diabetes | 0.3475 | 0.3223 |
| Prior dialysis | 0.5482 | 0.6242 |
| Number of years on dialysis | 0.1581 | 0.8806 |
| **Donor characteristics** | | |
| Age | 0.3419 | 0.3272 |
| Status of the donor (alive/dead) | 0.0522 | 0.1444 |
| Expanded donor criteria | 0.1364 | 1.0000 |
| **Transplant characteristics** | | |
| Delayed graft function | 0.7710 | 0.4527 |
| Length of hospital stay during transplantation | 0.1773 | 0.0500 |
| **Outcomes associated with early hospital readmission** |  |  |
| Death-censored graft failure within the first year after transplantation. | 0.1627 | 0.6015 |
| Mortality within the first year of renal transplant | 0.2280 | 0.6015 |

**Supplementary Table 4: Results of leave-one-out sensitivity analysis of the variables.**

| **Potential Associations** | **Pooled estimates** | **Lower limit 95% CI** | **Upper limit 95% CI** | **p value** | **Heterogeneity I^2^ (%)** | **Outlier study excluded** | **Pooled estimates and 95% CI (afterwards)** | **p value** | **I^2^ (%)** |
| --- | --- | --- | --- | --- | --- | --- | --- | --- | --- |
| **Recipient characteristics** | | | | | | | | | |
| Age | 2.05 | 0.90 | 3.20 | 0.0005* | 97 | Tavares MG et al. 2019 | 1.77 [0.89, 2.66] | < 0.0001* | 90 |
| Gender | 1.00 | 0.89 | 1.12 | 0.98 | 56 | Nguyen MC et al. 2020 | 1.01 [0.96, 1.07] | 0.66 | 5 |
| Black race | 1.31 | 1.11 | 1.55 | 0.001* | 64 | Nguyen MC et al. 2020 | 1.23 [1.08, 1.40] | 0.002* | 31 |
| Body mass index | 0.53 | -0.08 | 1.14 | 0.09 | 77 | Nguyen MC et al. 2020 | 0.24 [-0.36, 0.84] | 0.43 | 63 |
| Diabetes | 1.32 | 1.22 | 1.43 | < 0.00001* | 14 | Kang IC et al. 2018 | 1.34 [1.28, 1.40] | < 0.00001* | 0 |
| Prior dialysis | 1.32 | 0.98 | 1.78 | 0.07 | 57 | Nguyen MC et al. 2020 | 1.17 [0.83, 1.64] | 0.37 | 30 |
| Number of years on dialysis | 0.85 | 0.41 | 1.29 | 0.0001* | 99 | McAdams-Demarco MA et al. 2012 | 1.02 [0.82, 1.22] | < 0.00001* | 89 |
| **Donor characteristics** | | | | | | | | | |
| Age | 2.02 | 0.93 | 3.11 | 0.0003* | 96 | Tavares MG et al. 2019 | \| 1.81 [1.42, 2.20] \| \| --- \| | <0.00001* | 48 |
| Status of the donor (alive/dead) | 1.64 | 0.71 | 3.79 | 0.24 | 99 | McAdams-Demarco MA et al. 2012 | 1.42 [1.15, 1.76] | 0.0010  * | 68 |
| Expanded donor criteria | 1.35 | 0.81 | 2.25 | 0.25 | 93 | McAdams-Demarco MA et al. 2012 | \| 1.60 [1.11, 2.32] \| \| --- \| | 0.01* | 66 |
| **Transplant characteristics** | | | | | | | | | |
| Delayed graft function | 1.75 | 1.42 | 2.16 | < 0.00001* | 82 | Naylor KL et al. 2021 | \| 1.83 [1.63, 2.05] \| \| --- \| | < 0.00001* | 20 |
| Length of hospital stay during transplantation | 1.93 | 0.59 | 3.27 | 0.005* | 99 | Tavares MG et al. 2019 | \| 1.38 [0.91, 1.85] \| \| --- \| | <0.00001* | 89 |
| **Outcomes associated with EHR** | | | | | | | | | |
| Death-censored graft failure within the first year after transplantation. | 1.70 | 1.43 | 2.02 | < 0.00001* | 2 | - | 1.70 [1.43, 2.02] | < 0.00001* | 2 |
| Mortality within the first year of renal transplant | 1.46 | 1.27 | 1.67 | < 0.00001* | 0 | - | 1.46 [1.27, 1.67] | < 0.00001* | 0 |

OR: Odds ratio, CI: Confidence interval, p value: probability value, * statistically significant.

**Supplementary Figure 1:** PRISMAFlowcart summarizing the study selection process

**
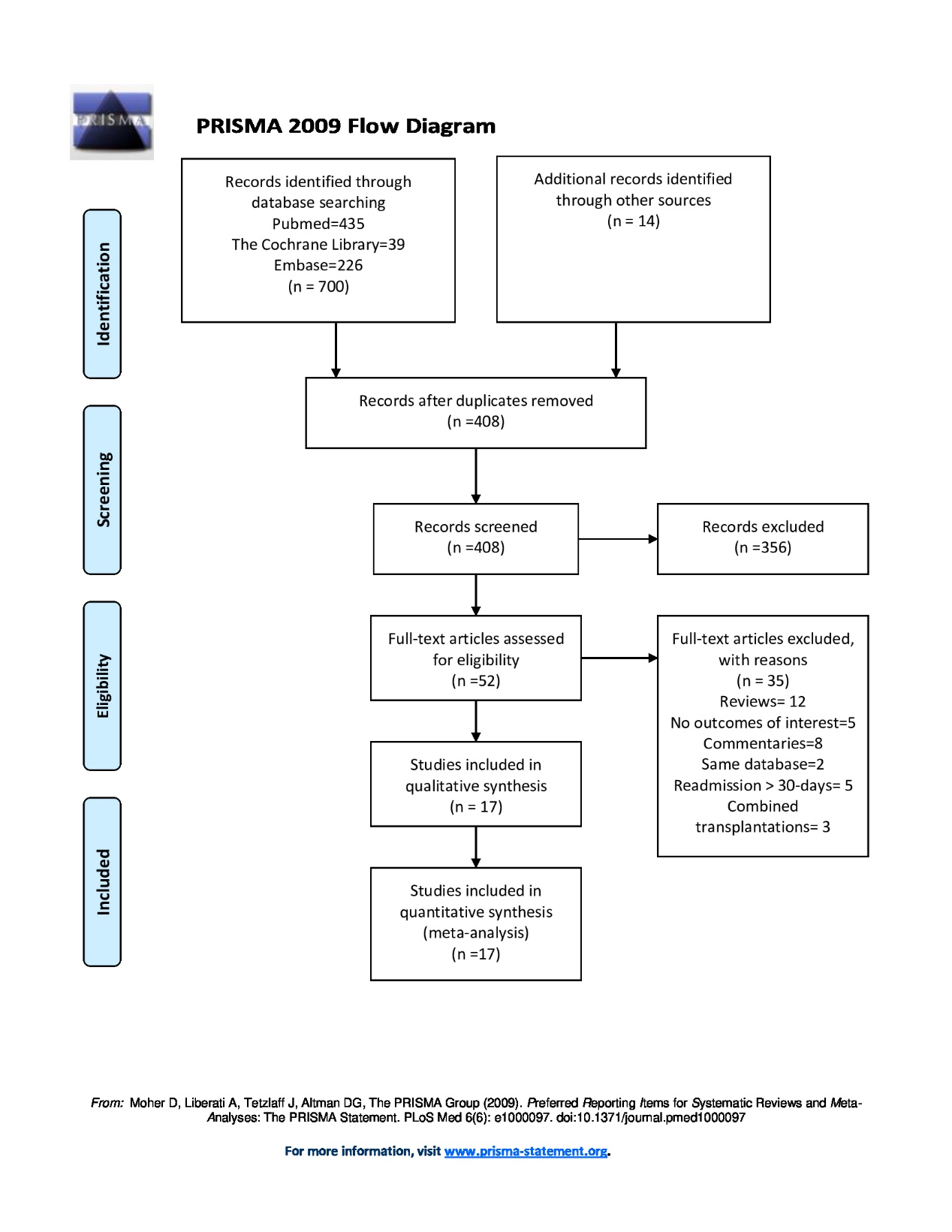
**

**Supplementary Figure 2:** Forest plot for the association of recipient’s age with Early hospital readmission.

**
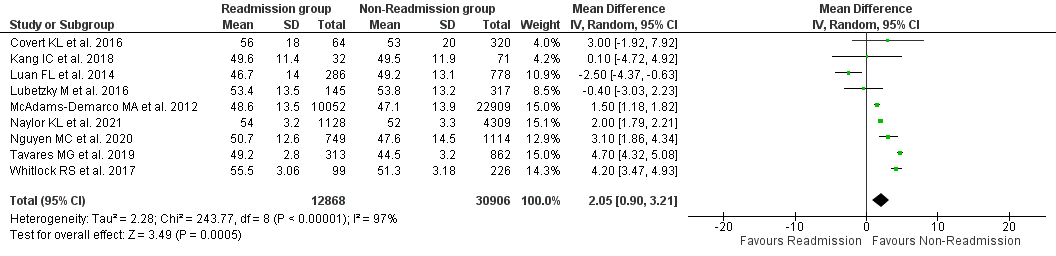
**

IV: inverse variance, SD: Standard deviation, CI: Confidence interval, p value: Probability value.

**Supplementary Figure 3:** Forest plot for the association of recipient’s black race with Early hospital readmission.

**
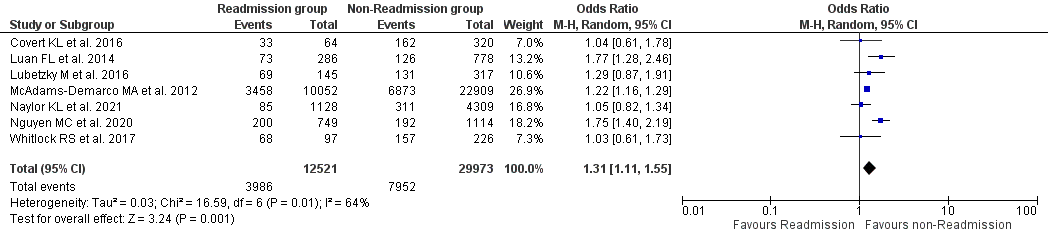
**

OR: Odds ratio, MH: Mantel Haenszel, CI: Confidence interval, p value: Probability value.

**Supplementary Figure 4:** Forest plot for the association of diabetic recipient with Early hospital readmission.

**
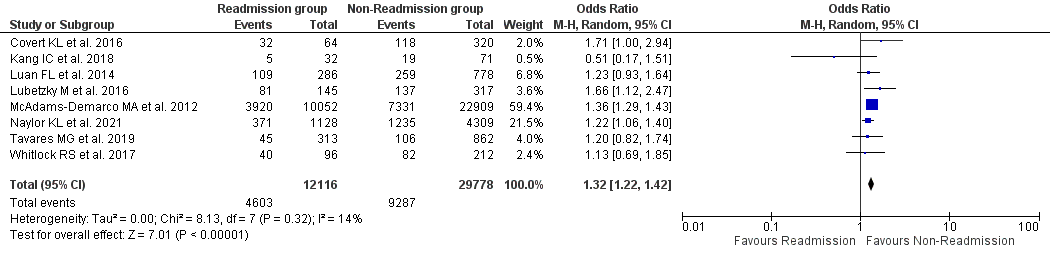
**

OR: Odds ratio, MH: Mantel Haenszel, CI: Confidence interval, p value: Probability value.

**Supplementary Figure 5:** Forest plot for the association of the number of years on dialysis with Early hospital readmission.

**
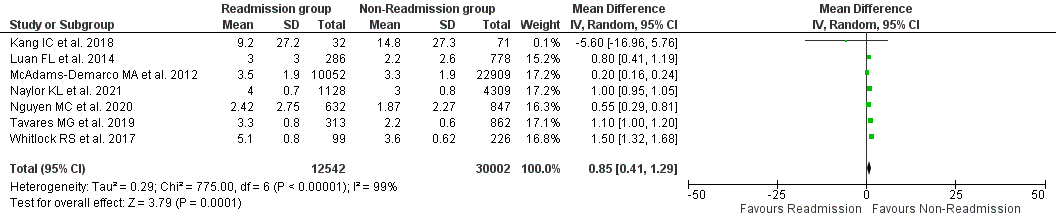
**

IV: inverse variance, SD: Standard deviation, CI: Confidence interval, p value: Probability value.

**Supplementary Figure 6:** Forest plot for the association of recipient’s male gender with Early hospital readmission.


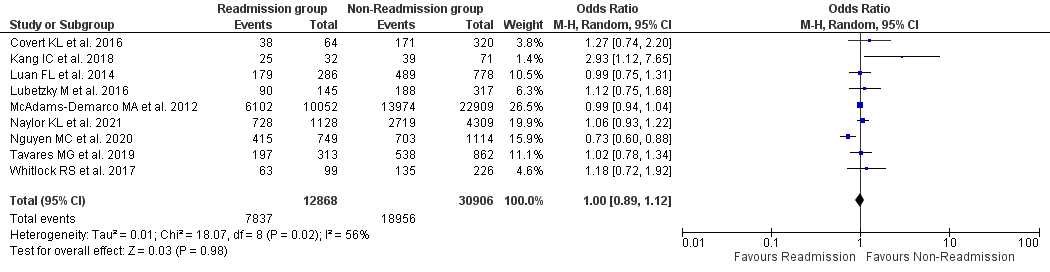


OR: Odds ratio, MH: Mantel Haenszel, CI: Confidence interval, p value: Probability value.

**Supplementary Figure 7:** Forest plot for the association of recipient’s body mass index with Early hospital readmission.

**
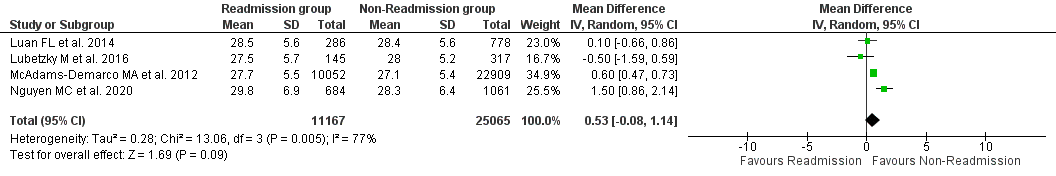
**

IV: inverse variance, SD: Standard deviation, CI: Confidence interval, p value: Probability value.

**Supplementary Figure 8:** Forest plot for the association of prior dialysis with Early hospital readmission.

**
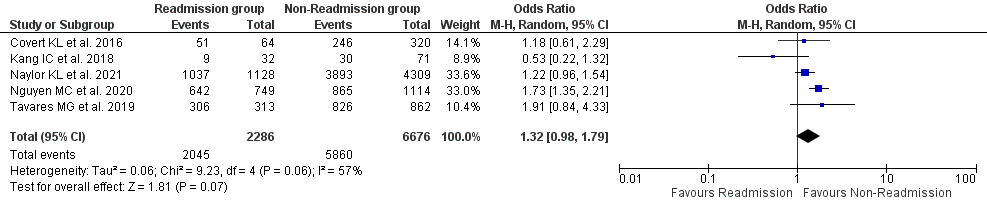
**

OR: Odds ratio, MH: Mantel Haenszel, CI: Confidence interval, p value: Probability value.

**Supplementary Figure 9:** Adjusted analysis for the association of recipient’s age with Early hospital readmission.


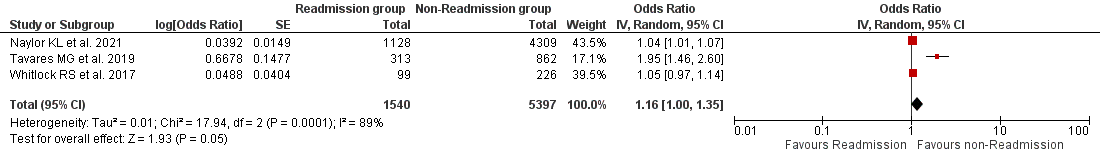


OR: Odds ratio, IV: inverse variance, CI: Confidence interval, p value: Probability value.

**Supplementary Figure 10:** Adjusted analysis for the association of the number of years on dialysis with Early hospital readmission.


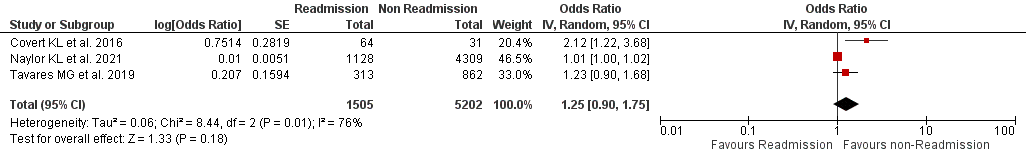


OR: Odds ratio, IV: inverse variance, CI: Confidence interval, p value: Probability value.

**Supplementary Figure 11:** Forest plot for the association of donor’s age with Early hospital readmission.


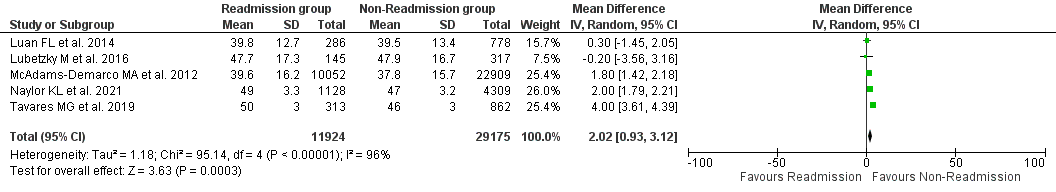


IV: inverse variance, SD: Standard deviation, CI: Confidence interval, p value: Probability value.

**Supplementary Figure 12:** Forest plot for the association of deceased donor transplant with Early hospital readmission.

**
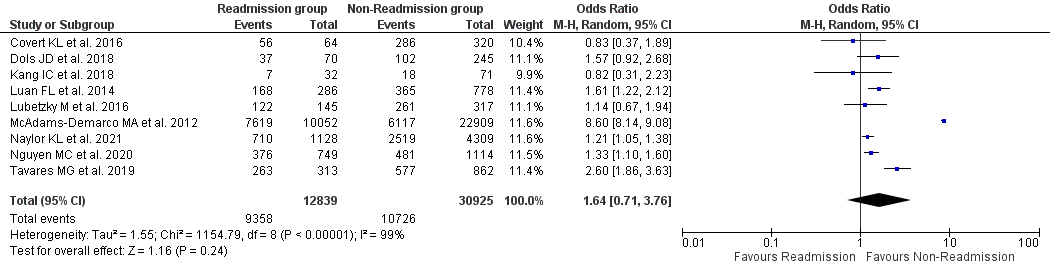
**

OR: Odds ratio, MH: Mantel Haenszel, SE: Standard error, CI: Confidence interval, p value: Probability value,

**Supplementary Figure 13:** Forest plot for the association of expanded donor criteria with Early hospital readmission.


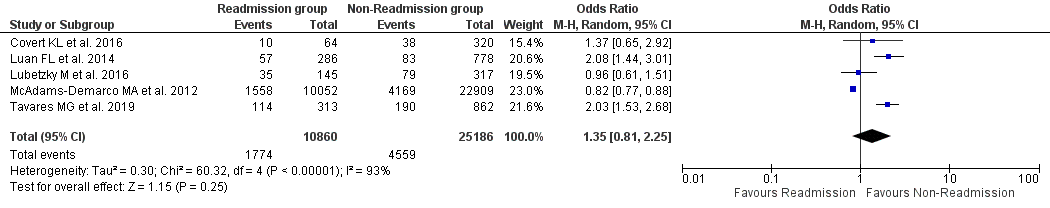


OR: Odds ratio, MH: Mantel Haenszel, SE: Standard error, CI: Confidence interval, p value: Probability value,

**Supplementary Figure 14:** Forest plot for the association of delayed graft function with Early hospital readmission.

**
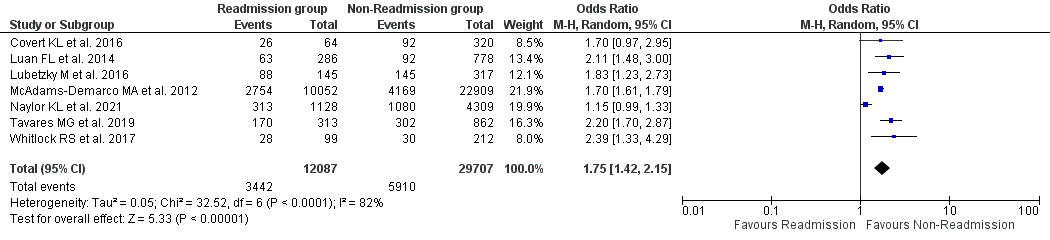
**

OR: Odds ratio, MH: Mantel Haenszel, SE: Standard error, CI: Confidence interval, p value: Probability value,

**Supplementary Figure 15:** Forest plot for the association of length of hospital stay during transplantation with Early hospital readmission.

**
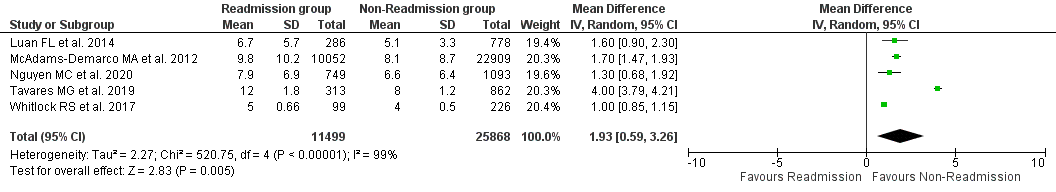
**

IV: inverse variance, SD: Standard deviation, CI: Confidence interval, p value: Probability value.

**Supplementary Figure 16:** Adjusted analysis for the association of length of hospital stay with Early hospital readmission.

**
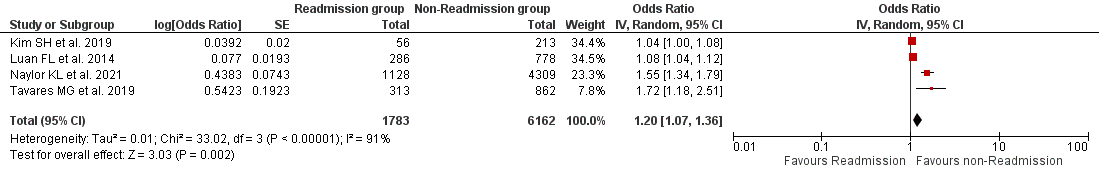
**

OR: Odds ratio, SE: Standard error, IV: inverse variance, CI: Confidence interval, p value: Probability value.

**Supplementary Figure 17:** Adjusted analysis for the association of delayed graft function with Early hospital readmission.

**
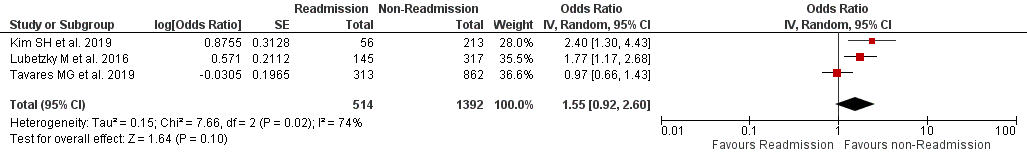
**

OR: Odds ratio, SE: Standard error, IV: inverse variance, CI: Confidence interval, p value: Probability value.

**Supplementary Figure 18:** Forest plot for the association of Early hospital readmission with death-censored graft failure within the first year after transplantation.


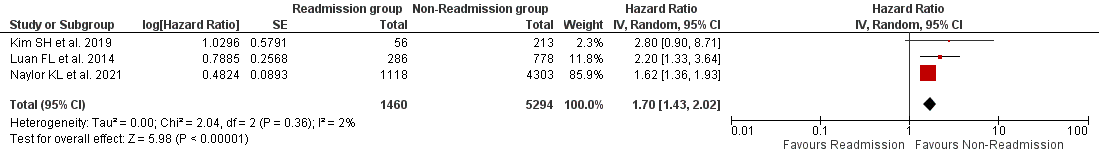


HR: Hazard ratio, SE: Standard error, IV: inverse variance, CI: Confidence interval, p value: Probability value.

**Supplementary Figure 19:** Forest plot for the association of Early hospital readmission with mortality within the first year of renal transplant

**
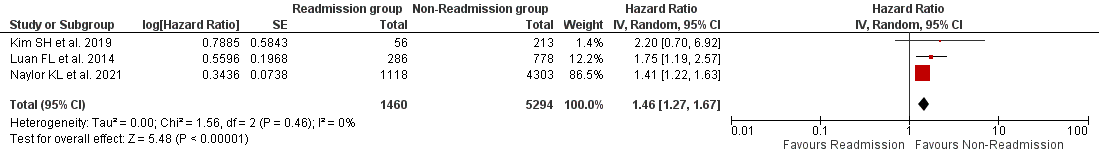
**

HR: Hazard ratio, SE: Standard error, IV: inverse variance, CI: Confidence interval, p value: Probability value.

**Supplementary Figure 20: Funnel plots for publication bias**

**
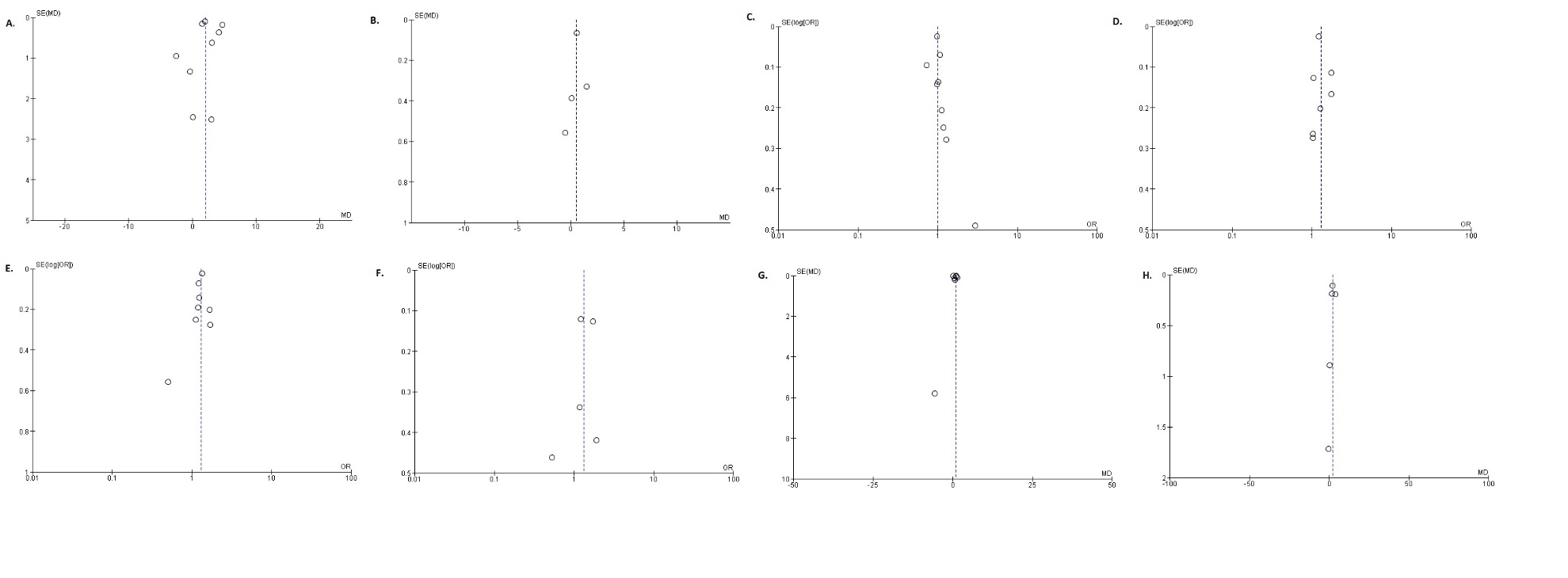
**

A. Recipient’s age. B. Recipient’s body mass index, C. Recipient’s gender, D. Recipient’s black race, E. Diabetes in recipient, F. Received prior dialysis, G. Number of years on dialysis, H. Donor’s age.

**Supplementary Figure 21: Funnel plots for publication bias**

**
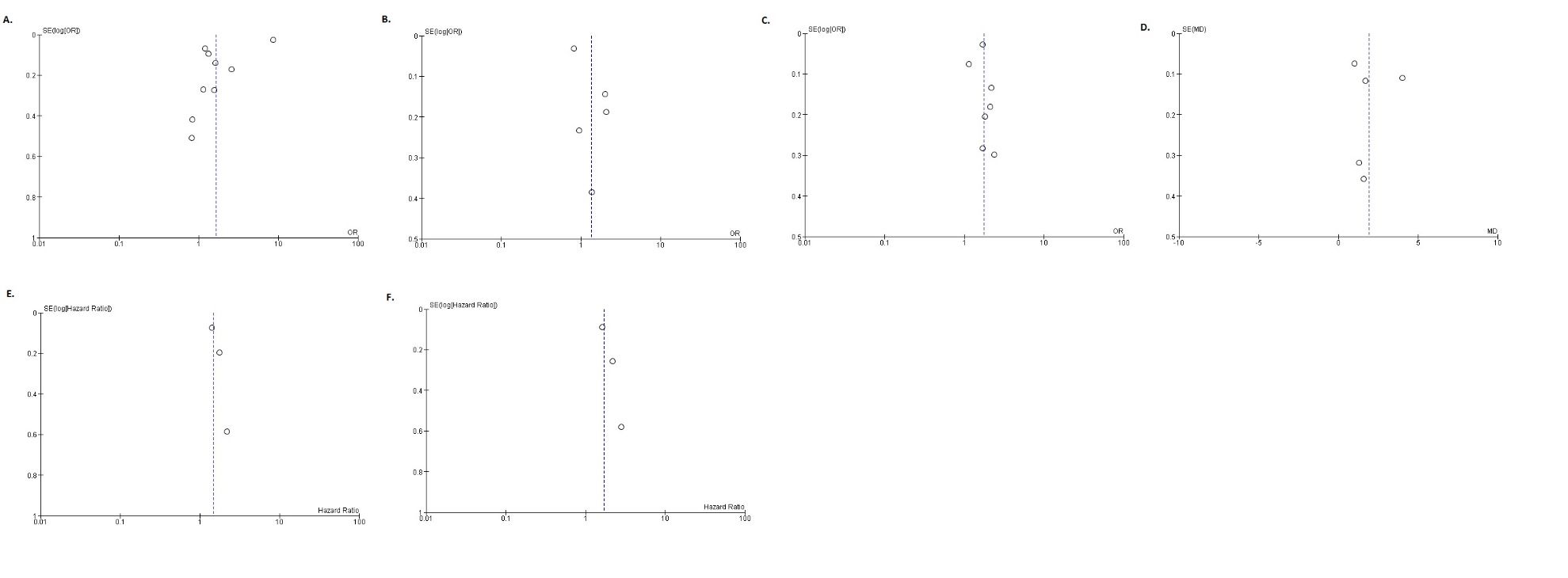
**

A. Deceased donor. B. Expanded donor criteria, C. Delayed graft function, D. Length of hospital stay, E. Mortality, F. Death-censored graft failure.
